# In Situ Observation of the Structure of Crystallizing Magnesium Sulfate Heptahydrate Solutions with Terahertz Transmission Spectroscopy

**DOI:** 10.1021/acs.cgd.2c00352

**Published:** 2022-05-20

**Authors:** Qi Li, Johanna Kölbel, Margaret P. Davis, Timothy M. Korter, Andrew D. Bond, Terrence Threlfall, J. Axel Zeitler

**Affiliations:** † Department of Chemical Engineering and Biotechnology, University of Cambridge, Philippa Fawcett Drive, Cambridge CB3 0AS, U.K.; ‡ Department of Chemistry, Syracuse University, 1-046 Center for Science and Technology, Syracuse, New York 13244, United States; ¶ Yusuf Hamied Department of Chemistry, University of Cambridge, Lensfield Road, Cambridge CB2 1EW, U.K.; § Department of Chemistry, University of Southampton, University Road, Southampton SO17 1BJ, U.K.

## Abstract

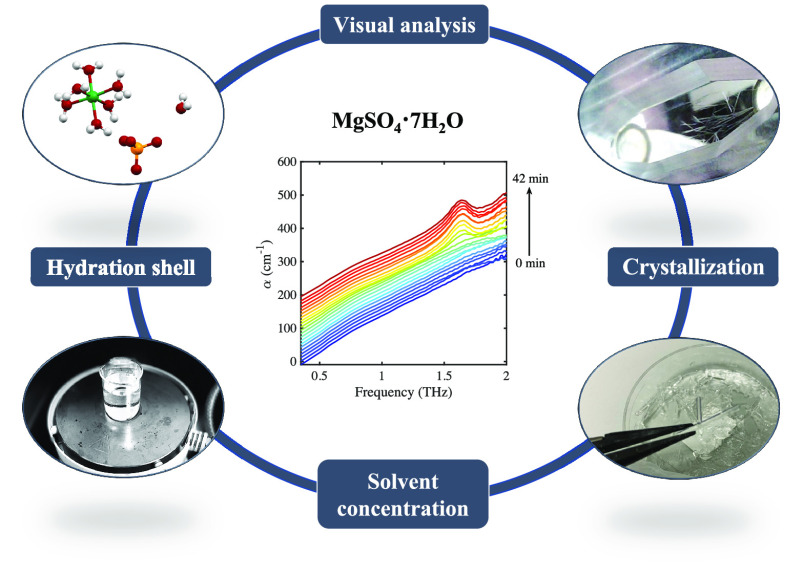

Terahertz time-domain spectroscopy in a transmission geometry combined with visual analysis was used to investigate the crystallization process of MgSO_4_ solution. Careful spectral analysis of both a feature at 1.6 THz and the overall magnitude of absorption allowed the extraction of information about the liquid phase before and during crystallization, aiding the investigation of solvation dynamics and the behavior of molecular species at phase boundaries. The method was reproducibly applied to a number of measurements on a series of solutions of three chosen concentrations at different temperatures. When increasing temperature at the end of the measurement, the dissolution of crystals was observed as well. The temperature-dependent absorption data of the semicrystalline systems were converted to the solvent concentrations using a recently developed method. Solutions of a series of concentrations were also investigated in the temperature range of 4–25 °C. The results were compared to the theoretical calculated values, and the consistent differences proved the existence of a hydration shell around the salt ions whose behavior is different from bulk water. Future work will focus on triggering nucleation at specific positions in order to study the very beginning of the crystallization process. MgSO_4_ heptahydrate is used as a model system in this study, while the concept and the setup can be applied to other systems.

## Abstract 2

Terahertz time-domain spectroscopy in a transmission geometry combined with visual analysis was used to investigate the crystallization process of MgSO_4_ solution. The results were compared to the theoretical calculated values, and the consistent differences proved the existence of a hydration shell around the salt ions whose behavior is different from bulk water.

## Introduction

The crystallization process has been used for centuries as a purification and separation step for various applications. Therefore, it is surprising that empirical models rather than fundamental understanding still govern the comprehension of crystallization’s underpinning mechanisms and kinetics. What is well-established is that nucleation and crystal growth are the two main steps contributing to the crystallization process. However, the microscopic mechanism of the formation of the nuclei and how they subsequently evolve into crystals is still unclear.^1−4^ Two widely popular models are used to describe the crystallization process: classical nucleation theory and nonclassical theory. The former states that density and order fluctuations in the solution cause the formation of crystal-like clusters, which in turn result in nuclei that gradually grow into the crystal form defined by the packing of the cluster.^5^ The nonclassical theory proposes that the clusters first formed are liquid like, and crystalline order is only introduced later when they grow into nuclei.^6,7^

One widely used model system for investigating crystallization is the MgSO_4_–H_2_O system. A variety of hydrate forms can crystallize depending on the temperature and concentrations in solution, but this system also recently received added attention, because the presence of such sulfates and their hydrated forms are discussed as the origin of near-surface water content on Mars.^8^ A comprehensive understanding of the crystallization mechanism is highly desirable to support further research into this topic.

A range of techniques are widely used to investigate the crystallization process: traditional crystallographic methods to characterize crystalline structures, such as small-angle and wide-angle X-ray scattering, X-ray spectroscopy, and X-ray diffraction; spectroscopic techniques including nuclear magnetic resonance (NMR), Fourier transform infrared spectroscopy (FTIR), and Raman spectroscopy to provide insight into the chemical structure and the shape of the molecules during crystallization; and UV–vis spectroscopy to measure the solute concentration (but this is of limited use when nascent particles result in scattering losses that cannot be distinguished from absorption).^3,9^ Turbidimetry is used to measure the loss of intensity of transmitted light due to the scattering effect of those particles. Second-harmonic generation and polarized light microscopy are applied to detect the onset of crystallization but are not very sensitive to the structure.^10,11^

When investigating crystallization in aqueous solutions, the strong absorption due to the presence of water makes it difficult to perform FTIR measurements in transmission. Instead, observations are restricted to surface measurements using attenuated total reflection (ATR). While Raman spectroscopy does not suffer from this restriction, the lack of interaction means that only very little information, if any, of the solvent molecules during the crystallization process can be extracted.^12^

Terahertz time-domain spectroscopy (THz-TDS) offers a unique perspective to characterize the crystallization process both in terms of information from the solvent as well as the emerging crystals. By measuring the amplitude and phase of single-cycle pulses of far-infrared radiation, in solution, THz-TDS can probe large-amplitude intermolecular vibrations as well as high-frequency dielectric relaxation processes that correspond to relaxation times of less than 10 ps.^13^ In solids, the technique can distinguish between different polymorphic forms, cocrystals, hydrates, and solvates as well as provide an excellent measure for overall crystallinity and defect density crystallinity, since the long-range order in crystals results in well-defined spectral features (fingerprints) in the terahertz region. In contrast, in amorphous materials, the lack of long-range order results in the collapse of the well-defined peaks into a vibrational density of states (VDOS), which starts at a few hundred gigahertz and exceeds the entire spectral bandwidth of many THz-TDS spectrometers (0.3 to 3 THz). It is characterized by a featureless, monotonously increasing absorption coefficient that typically peaks at frequencies beyond 3 THz.^14^

The contributions of individual atomic motions in experimental terahertz spectra are not discernible without additional information, which is usually gathered from theoretical simulations. Density functional theory (DFT) simulations provide normal mode vectors and force constants and can therefore be used to investigate and visualize vibrational modes.^15^

Studies of water and water/alcohol mixtures with THz-TDS suggested key concentration transition points that marked different stages of water and alcohol molecular interactions.^16^ Other water mixtures and solutions also demonstrated the use of THz-TDS to probe water molecules based on their mobilities and the behavior of the hydration shell.^17,18^ As well as for characterizing static structures, THz-TDS has also been found useful to probe reaction dynamics, such as solid–solid phase transitions, amorphous–solid transformations, and crystallization.^13,19−21^

Previously, terahertz spectroscopy has been applied to study the crystallization of sugar and l-(+)-tartaric acid utilizing attenuated total reflectance geometry and triggering the crystallization process by the evaporation of water from the aqueous solution.^20,21^ In a separate experiment, terahertz narrow-band absorption and time-domain spectroscopy were combined to investigate the early stages of CaCO_3_ nucleation. Experimental evidence for the nucleation to occur via the prenucleation pathway for aqueous systems was found.^22^

During crystallization, the VDOS is depleted, resulting in the emergence of peaks and a dropping of the overall baseline given its nature as the flank of the VDOS. It is important to emphasize that this change in the spectral response of the absorption baseline in the frequency range studied is not the result of a drift in the background signal but contains quantitative information regarding the depletion of the VDOS as well as the dielectric relaxation dynamics on picosecond to femtosecond time scales. THz-TDS can therefore simultaneously probe amorphous and crystalline phases represented by the behavior of the baseline and peaks, respectively. In addition, this suggests that the behavior of the liquid phase can be extracted from the baseline while crystallizing; hence, the solute concentration can be measured even in semicrystalline samples.^23^

MgSO_4_ is chosen as a model system to demonstrate that THz-TDS is an option to complement the currently widely applied tools in the field of crystallization. The versatile setup based on THz-TDS in transmission geometry and the methodology described in detail in refs (24) and (23) is further used to observe the dissolution of crystals at elevated temperatures as well as calculate the equivalent local concentration and can be extended to other systems of interest.

## Methods

### Solid-State Sample Measurements

Commercial samples of four different MgSO_4_ hydrates were investigated (as listed in Table S1 in the SI). Powder X-ray diffraction measurements were made on a Panalytical XPert Pro diffractometer in Bragg–Brentano geometry using nonmonochromated Cu Kα radiation (λ_ave_ = 1.5418 Å). Samples were prepared on glass flat-plate sample holders, and data were measured over the range 2θ = 5 to 70° with an effective step size of 0.0167° and counting time of 60 s per step. Measured data were compared to simulated patterns generated using Mercury^25^ from available crystal structures of MgSO_4_,^26^ MgSO_4_·H_2_O,^27^ and MgSO_4_·7 H_2_O.^28^

For terahertz measurements, the crystalline samples were ground gently in an agate mortar with a pestle, and the polycrystalline samples were then mixed with polyethylene (Induchem, Volketwil, Switzerland) to a defined concentration that varied for different hydrates, as detailed in Table S1 in the SI.

The well-mixed powder was compressed into a pellet of 13 mm diameter with a thickness of 2 to 3 mm using a hydraulic press (Specac Ltd., Kent, UK) at a load of 2 ton, and a blank polyethylene pellet prepared in the same way was used as a reference. During the THz-TDS transmission measurement, 1000 waveforms were acquired and averaged, with a resolution of 0.94 cm^–1^.

Additionally, a supersaturated solution of MgSO_4_ was prepared from MgSO_4_ heptahydrate 98% (Sigma-Aldrich, Gillingham, UK) dissolved in Milli-Q water (IQ 7000, Merck, Darmstadt, Germany, resistivity 18.2 MΩ·cm) and filled into a well-sealed Petri dish. The Petri dish was left in a fume hood at 20 °C until crystals formed. This process was to mimic the crystallization process in the crystallization cell. Due to constraints of the setup, it was impossible to investigate crystals grown directly in the microfluidic cell at cryogenic temperatures. The crystals grown in the Petri dish were made into pellets using the method described above and characterized at cryogenic temperatures with terahertz spectroscopy later to confirm their structure more accurately.

THz-TDS measurements were performed with a commercial spectrometer TeraPulse 4000 (TeraView Ltd., Cambridge, UK), and the measurement chamber was purged with nitrogen to eliminate the effect of water vapor. Variable-temperature measurements were facilitated by a cryostat (Janis, Massachusetts, USA), and the temperature was well-controlled with an attached temperature controller Lakeshore 330 (Ohio, USA). The sample pellets were first cooled down to 80 K and then heated up in steps to 300 K to examine the temperature-dependent behavior of the spectral features. Measuring commercial samples at room temperature allows the direct comparison of their spectra with those acquired during crystallization experiments. However, acquiring spectra for crystalline samples at lower temperatures improves their quality, since absorption in the terahertz region is highly affected by the temperature background, reflected in effects such as peak broadening and peak shifting.

### Computational Methods

The solid-state density functional theory (ss-DFT) program CRYSTAL17^29^ was used to perform geometry optimization and frequency analysis calculations on crystalline MgSO_4_ heptahydrate using periodic boundary conditions. All calculations utilized the revised version of the Peintinger–Oliverira–Bredow split-valence triple-ζ basis set (pob-TZVP-rev2)^30^ and the Becke–3-Lee–Yang–Parr (B3LYP)^31,32^ hybrid density functional. The B3LYP density functional was supplemented with Grimme’s noncovalent dispersion correction (D3) and the Becke–Johnson damping correction^33−35^ with three-body Axilrod–Teller–Muto repulsion contributions (program keyword “ABC”).^36−38^ In all MgSO_4_ heptahydrate calculations, 125 k-points were used in the irreducible Brillouin zone (keyword SHRINK*=*9), and 99 radial point and 1454 angular points were used for the pruned integration grid. The overlap-based truncation criteria for the bielectronic integrals (Coulomb and exchange) (program keyword “TOLINTEG”) were set to 10^–12^, 10^–12^, 10^–12^, 10^–20^, and 10^–40^ for all calculations, and the maximum order of multipolar expansion was set to 6 (program keyword “POLEORDR”). The starting structure for MgSO_4_ heptahydrate was published by Ferraris, Jones, and Yerkess in 1973,^28^ and the initial ionic charges were explicitly set to Mg^2+^ and (SO_4_)^2–^. In the geometry optimization, the lattice dimensions and atomic positions were allowed to fully optimize within the *P*2_1_2_1_2_1_ space group (Schoenflies symbol: *D*_2_^4^), and the energy convergence was set to *ΔE* < 10^–8^*E*_h_. The optimized structure was used to calculate the vibrational frequency analysis, and the energy convergence was set to *ΔE* < 10^–10^*E*_h_. The vibrational frequency analysis determined that the optimized structure was a minimum on the potential energy surface (no negative frequencies). During the frequency analysis, each atom was displaced twice along each Cartesian axis, and the numerical derivatives of the Hessian matrix were calculated using the central difference formula. The IR intensities were calculated using the Berry phase method.^39,40^

### Crystallization Measurements

To investigate crystallization, magnesium sulfate solutions were prepared at various concentrations using commercial MgSO_4_ heptahydrate 98% (Sigma-Aldrich, Gillingham, UK). The sample was dissolved in Milli-Q water (IQ 7000, Merck, Darmstadt, Germany, resistivity 18.2 MΩ · cm) in a beaker, which was then left on a magnetic stirrer until the crystals were fully dissolved. After several rounds of preliminary experiments, three concentrations (mass ratio of MgSO_4_ heptahydrate to water) were chosen for further repeats: 1.41:1, 1.29:1, and 1.20:1, corresponding to molar ratios of 0.103:1, 0.094:1, and 0.088:1, respectively. This was based on the time and temperature observed for crystallization.

A detailed description of the crystallization setup was given in an earlier paper.^24^ The setup consisted of a liquid cell (thickness 100 μm) that was held by a hollow metal sample holder, inside of which water was circulated. The temperature of the circulating water was controlled via an external water bath, and its temperature was balanced between an electric heater and a surrounding ice bath with an accuracy of 0.1 °C. The operation temperature was in the range of 4 to 90 °C, and during the experiments described here, it was operated between 4 to 25 °C.

The temperature during measurements was recorded independently at three different positions in the setup. It was not possible to measure the actual temperature inside the liquid between the spacers of the cell due to space constraints, but one reading was taken in immediate proximity. The temperature measurement instrument had a resolution of 0.025 °C for the Type K thermocouples used. The thermal mass of the metal block that was attached to the quartz cell and that was used to circulate the water through was much larger than that of the crystallization cell, and any potential temperature difference would therefore be negligible in the context of this experiment.

Air was used as the reference, and the high-resolution mode of the spectrometer was utilized to extend the extent of acquired time-domain waveforms to 45 ps. Each spectrum was formed of the average of 15 individual waveforms with a spectral resolution of 0.94 cm^–1^, resulting in an acquisition time of 20 s per spectrum. The valid frequency range was from 0.35 to 2 THz.

When monitoring the crystallization process at a set temperature, the cell was first cooled down and kept constant at the target temperature until the system was stable. Afterward, the MgSO_4_, solution was injected into the flow cell with a syringe via a tube, and the outlets on both sides of the flow cell were sealed with parafilm. The sample holder including the cell was promptly placed at the center of the measurement chamber, and terahertz spectra and images were acquired. The time from injecting the solution to the start of the measurement was minimized to no more than 30 s, in case of triggering undesired nucleation. The temperature was kept as constant as possible during the whole crystallization process, until crystals formed across the cell in the view of the optical probe and the terahertz spectra did not exhibit further changes. The experiment was either terminated at this point, or the behavior of the system during slow heating to room temperature was studied. In the latter case, the temperature was increased by 0.2 °C min^–1^ up to 25 °C in the flow cell. This was found to be an ideal heating rate to introduce a constant temperature change to the cell.

For all measurements, three thermocouples monitored the temperature at various positions: in the water bath, inside the metal sample holder, and at the inlet of the cell, and one data point was acquired per second. The optical probe used for image acquisition was set to acquire one photograph every two seconds. The time-stamped images of each measurement were further analyzed using ImageJ. Edge detection was performed using a Sobel edge detector to highlight intensity changes.^49^ The images were then binarized (using the same threshold settings for all images), and the background was set to black. Crystalline features were then represented by white pixels, which were counted using the “Measure” functionality of ImageJ. The area fraction of white pixels was normalized to 0 (at the beginning of the measurement) and 100 (once crystals had covered the whole cell) and was linked to the time of acquisition and hence terahertz measurements. It was observed that a sigmoid described the process well. After each crystallization measurement, the liquid cell was thoroughly cleaned to remove grown crystals, contaminations, or seeds, which could influence subsequent measurements. The cleaning solution was prepared from commercial EDTA solution (pH = 8; Fisher Scientific, Loughborough, UK) and NaOH solution (Reagecon Diagnostics, Shannon, Ireland) to adjust the pH to 10, in which MgSO_4_ exhibits a higher solubility.

## Results and Discussion

### PXRD Analysis of MgSO_4_ Hydrates

Commercially available samples of anhydrous MgSO_4_ from two different suppliers and the MgSO_4_·7 H_2_O sample were highly crystalline and agreed closely with the patterns simulated from the crystal structure.^26,28^

The MgSO_4_·H_2_O sample showed much broader peaks, indicative of a smaller particle/domain size. It largely agreed with the pattern simulated from the monohydrate crystal structure,^27^ but additional peaks at 2θ ≈ 20, 32, and 40° (marked by an asterisk in Figure S2 in the SI) indicated the presence of an additional minor phase. Comparison to other known MgSO_4_/H_2_O phases suggests the impurity was most likely to be hexahydrate:^41^ its most prominent peak matched that seen at 2θ ≈ 20°, plus groups of peaks just above 30° 2θ and just below 40° 2θ could match to the features seen in the monohydrate sample.

All measured and simulated PXRD patterns can be found in the Supporting Information.

### Terahertz Spectra of MgSO_4_ Hydrates

**Figure 1:**
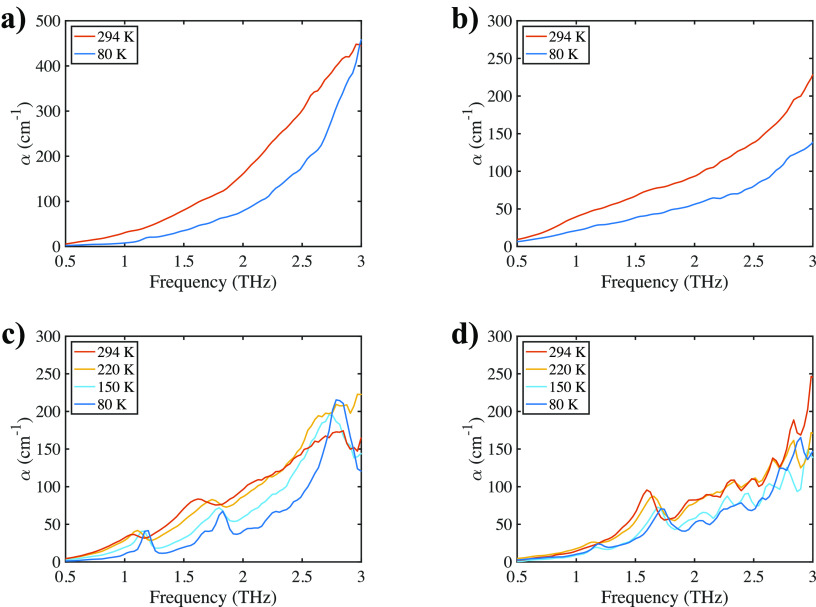
Crystalline MgSO_4_ hydrates measured at different temperatures: (a) anhydrous MgSO_4_; (b) MgSO_4_ monoydrate; (c) commercial MgSO_4_ heptahydrate; and (d) MgSO_4_ heptahydrate grown in the lab from solution.

The terahertz spectra of MgSO_4_ anhydrous, monohydrate, and heptahydrate as well as the crystal grown in the lab from solution were acquired at different temperatures (Figure 1). Comparing the spectra of the three different hydrates, neither the anhydrous nor monohydrate forms of MgSO_4_ showed pronounced peaks in the region of interest (0.3 to 3.0 THz), and the only change that was observed upon cooling was a drop in the baseline.

For the commercial heptahydrate sample, three pronounced bands were observed at 80 K: at 1.2 THz, 1.7 THz (double features), and 2.8 THz. These vibrations probably resulted from the interactions between MgSO_4_ and water, because they were not present in the anhydrous and monohydrate samples. As expected, the spectra exhibited peak broadening and shifting as well as an increase of the baseline upon heating to room temperature. This is due to the significant population of excited vibrational states at room temperature, which is characteristic of the far-infrared where the energy gap between ground state and excited states is on the order of several meV and therefore slightly lower and close to *k*_b_T at room temperature. In addition, the increased thermal vibration and emission contribute to this effect. At 294 K, which was close to the temperature of the crystallization experiments, the high-intensity peak at 2.8 THz diminished into the baseline, and the two features at lower frequencies became weaker and broader, while the double peak at 1.7 THz merged and shifted to a single feature at 1.6 THz.

The spectra of crystals grown under the conditions similar to the crystallization in the flow cell exhibited less temperature-dependent behavior. The feature at 1.2 THz was slightly more intense at low temperatures, while the peak at 1.7 THz was consistently observable in the whole temperature range. The latter shifted to 1.6 THz upon heating to room temperature, though as a single feature rather than a double one at temperatures above 80 K. The high similarity between the terahertz spectra of heptahydrate and grown crystals, especially at 294 K, confirmed that the crystals grown in the crystallization cell were indeed MgSO_4_ heptahydrate. The differences between the two could be accounted for by the different purities and defect densities. In addition, the 1.1 THz peak became too weak to be observed at room temperature, so the feature at 1.6 THz was used in the following analysis to monitor the crystallization process.

**Figure 2:**
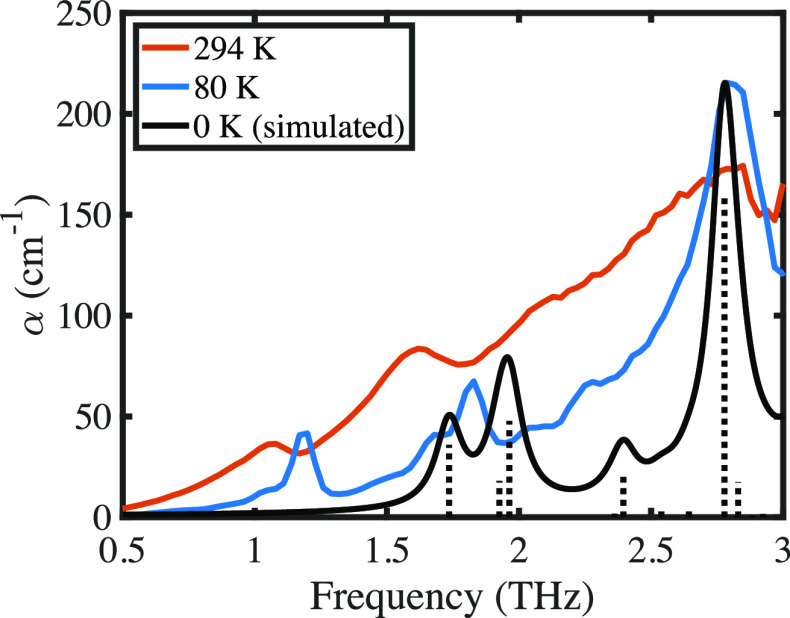
Comparison of simulated (black) and measured spectra of MgSO_4_ heptahydrate. Simulated data have been scaled to the feature at 2.8 THz. Dotted lines denote positions and relative intensities of infrared-active modes.

Within the inherent limitations of the computational methodology,^42^ the ss-DFT simulation produced a good correlation with the experimental results (see Figure 2). The relative shift in the frequencies of the features between calculation and experiment is expected due to the difference in temperature between calculation and the experimental data among other factors. No scaling was applied to the frequency of the calculated modes. The calculation revealed that the double features near 1.7 THz originate from three distinct lattice vibrational motions predicted to be at 1.74, 1.93, and 1.96 THz. As outlined above, the slight overestimation of the vibrational frequencies is attributable to the simulation being performed at 0 K, while the experimental data is acquired at temperatures ≥80 K. The predicted 1.74 THz vibration (B3 symmetry) involves primarily the rotational motions of the [Mg(H_2_O)_6_]^2+^ and (SO_4_)^2–^ moieties about the *c*-axis of the crystallographic unit cell, with a smaller contribution of translational motion along the *a*-axis. The atomic motions associated with the optical phonons observed in this work preserve the center of mass of the unit cell. This is achieved by the different unit cell components moving in specific phase relationships to one another as can be seen in the provided vibrational mode animations (see the Supporting Information). The 1.93 THz vibration (B3 symmetry) is a translational vibration of the crystal components along the *c*-axis. The 1.96 THz mode (B2 symmetry) is largely rotational motion like the 1.74 THz mode but with rotation about the *c*-axis and some translational motion along the *b*-axis. The intense experimental peak near 2.8 THz is predicted at 2.78 THz (B3 symmetry) and is a rotational lattice vibration about the *a*-axis with a small component of translation along the *c*-axis. Noticeably missing from the simulation is a feature matching with the experimental peak at 1.2 THz, as the 1.74 THz vibration is the lowest frequency vibration (infrared or Raman) produced by ss-DFT. The reason behind this absence is not clear. The use of other basis sets and density functionals did not produce the lower feature nor did the explicit calculation of transverse optical (TO) and longitudinal optical (LO) phonon splitting. One possible explanation is that the published space group of *P*2_1_2_1_2_1_ is not an accurate representation of the symmetry of the crystal at reduced temperatures and is instead *P*2_1_ (Schoenflies symbol: C_2_^2^) as suggested by others.^43^ A reduction in crystal symmetry may yield new vibrations in the ss-DFT predicted spectra, but such simulations are not trivial given the high computational cost of the much larger asymmetric unit.

### Crystallization of MgSO_4_·7H_2_O

**Figure 3:**
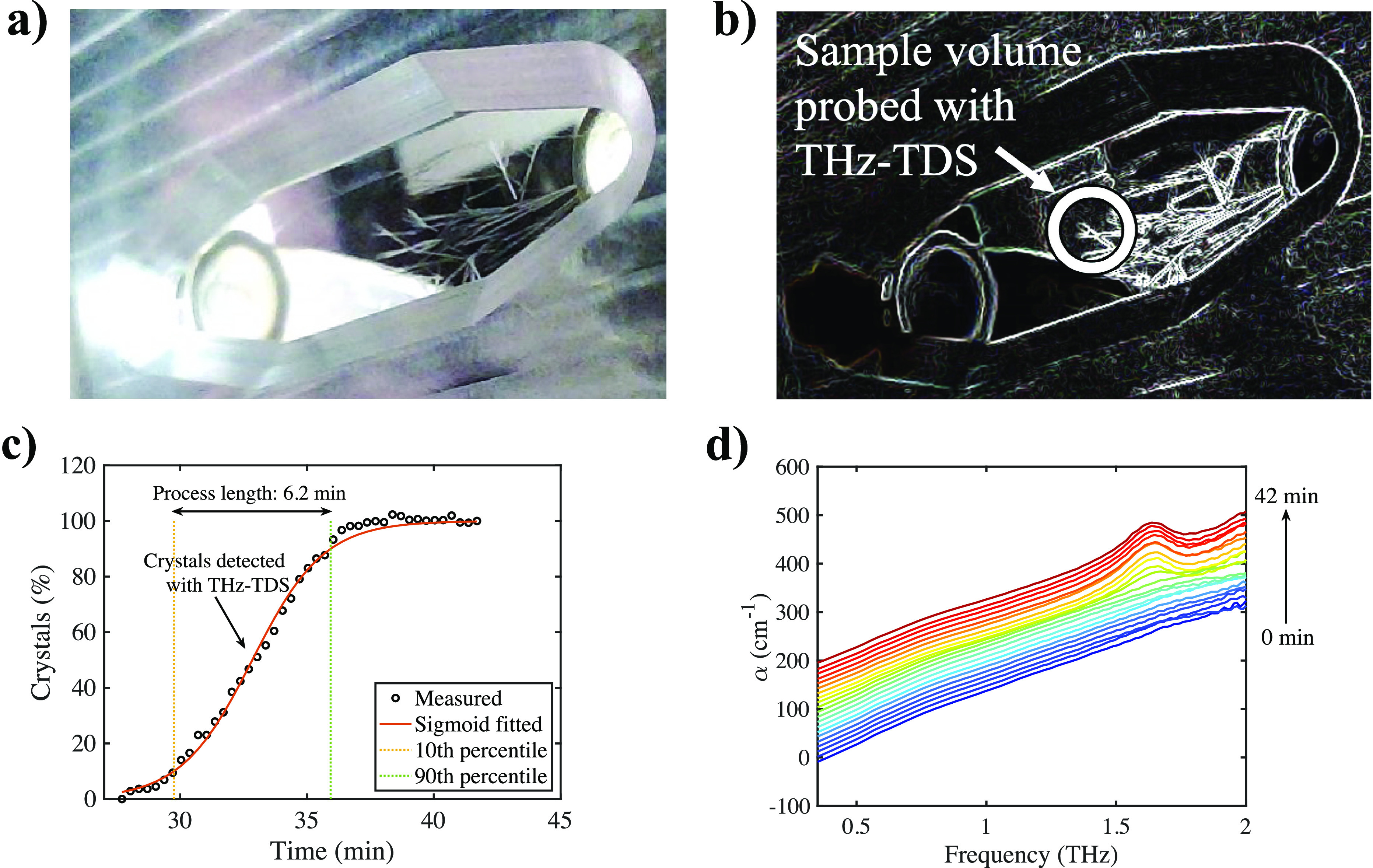
Visual analysis of crystal growth. (a) Raw image recorded when the crystals grew into the middle of the cell and were detected by THz-TDS. (b) Same image after edge detection. The approximate sample volume probed with THz-TDS is highlighted. (c) Percentage of area covered by crystals as observed with visual analysis plotted against time. In this case, the crystal growth through the cell occurred in about 6 min at around 4 °C. (d) Terahertz spectra acquired during crystallization. Each subsequent spectrum is offset by 10 cm^–1^.

As described in the method section, the flow cell was kept constant at the desired temperature for crystallization, and the process was monitored with both terahertz spectroscopy and an optical probe. Confirmed by visual analysis, crystallization was usually observed to start at either inlet or outlet (or both) of the crystallization cell, followed by crystal growth across the cell to its other end. Acquired images were useful complementary information to track the progress of crystal growth throughout the cell. After performing image edge detection and binarization, crystals were represented by white pixels, and the amount of crystals in the field of view of the camera was quantified as demonstrated in Figure 3. In most measurements, it took approximately 4 to 10 min for crystals to grow from one end of the cell to the other once crystal growth had initiated.

The spot of terahertz radiation probing the center of the cell was about 2 mm in diameter (as highlighted in Figure 3b). Before the crystals had grown into the center of the cell, the sample volume probed with terahertz radiation was entirely filled with liquid, and the terahertz spectra were hence completely featureless. However, as crystal growth continued toward the center of the cell, the absorption below 1.6 THz decreased, and a peak emerged at 1.6 THz. This indicated the existence of crystals in the field of view of the spectrometer (see Figure 3d). The peak at 1.6 THz correlated with the peak in the solid-state heptahydrate samples measured previously and shown in Figure 1. The time by which crystals were detected by THz-TDS coincided well with the time expected from image analysis (also shown in Figure 3c).

**Figure 4:**
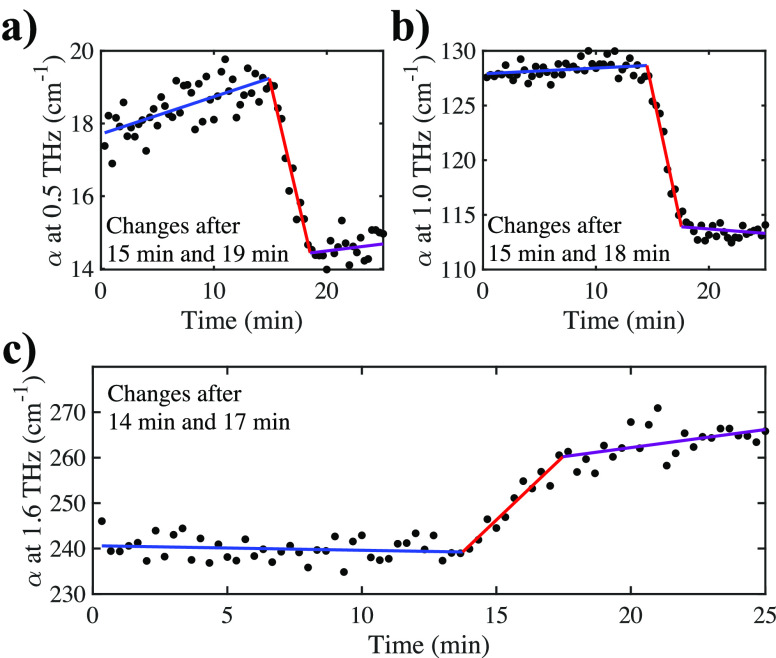
Absorption extracted at 0.5, 1, and 1.6 THz. This highlighted the different behaviors of the peak feature compared to the rest of the spectrum (i.e., the differences between crystalline and liquid phases). Whereas the absorption at 1.6 THz increased after 14 min, when crystallization occurred, the absorption decreased at lower frequencies. Linear fits were performed before (first region, blue), during (second region, red), and after (third region, purple) crystal growth through the field of view.

Three frequencies were chosen to illustrate the changes of the spectrum over the course of the experiment: 1.6 THz, i.e., the peak maximum, 1.0 THz, the frequency where the spectrometer has the highest signal-to-noise ratio, and 0.5 THz, which was a sufficiently low frequency that it should not directly be influenced by the crystalline spectral feature. At each of those three frequencies, the absorption coefficient was extracted and plotted as a function of time, as illustrated in Figure 4.

An algorithm was used to differentiate reliably and reproducibly between three regions (before, during, and after crystal growth) by fitting three linear functions to the data and selecting the fits that minimized the sum of their root-mean-square error. The code is based on an algorithm previously used to identify glass transition temperatures from THz-TDS data.^50^ The fits are in Figure 4. This allowed more information to be extracted at each stage of the crystallization and facilitated comparison between the subsequent measurements, which were performed under a range of conditions. The variation in absorption coefficient between two subsequent points is on the order of 1 to 2 cm^–1^ at 0.5 THz, whereas the observed step height was about 5 cm^–1^ at 0.5 THz and larger at higher frequencies. Due to the time scale of the experiment, enough data points were available to perform a linear regression to observe clear trends. The random error in each measured data point was caused by a combination of power fluctuations, waveform averaging, and changes in the sample during the acquisition time (e.g., 20 s).

Crystallization experiments and the analysis described above were performed for a range of different temperatures and concentrations. A measure for how fast the crystals covered the field of view was found by evaluating the time difference between the emergence of the peak and reaching the equilibrium afterward. This time period was denoted as “second region”. The “first region” corresponded to the time before crystals appeared in the field of view, and the “third region” referred to the last part of the experiment after crystals had fully covered the field of view.

During analysis, the slope of the linear fit to the data points in the second region was evaluated. In most experiments, the gradient of the linear fit was positive for 1.6 THz and negative at lower frequencies.

**Figure 5:**
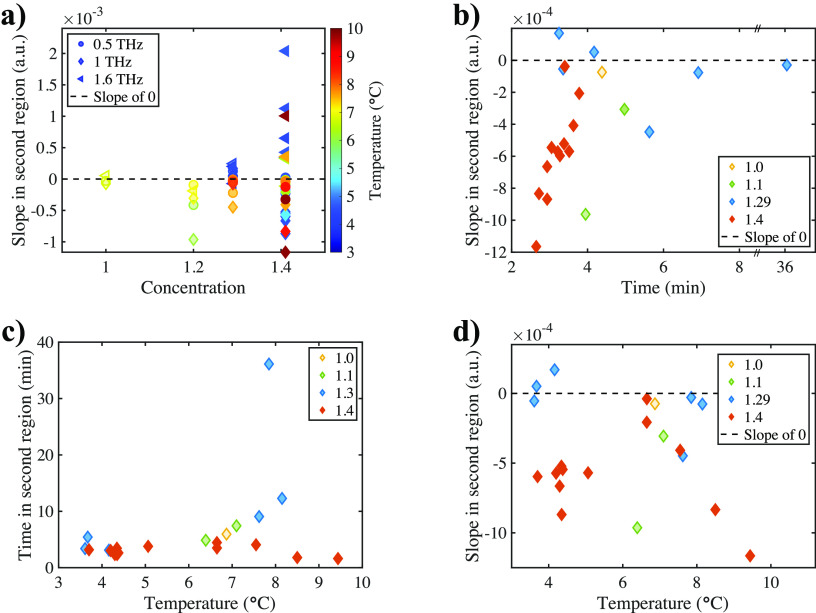
Analysis parameters during crystal growth plotted against concentration, time, and temperature. The concentrations were represented by the mass ratio of MgSO_4_ heptahydrate to water. (a) Slope in the second region plotted against concentration, shown at 0.5 THz (dots), 1.0 THz (diamonds), and 1.6 THz (triangles). The color denotes the temperature at which the system was kept during crystallization. (b) Gradient of the linear fit to the absorption at 1.0 THz in the second region plotted against time. The colors denoted the different initial concentrations. (c) Time spent in the second region plotted against temperature. The data was extracted at 1.0 THz, and different colors denoted different initial concentrations. (d) Slope in the second region plotted against the temperatures at which the experiments were performed, shown at 1.0 THz, and different colors denoted different initial concentrations.

In Figure 5, a range of experiments are presented that systematically explore the important factors during crystallization, such as temperature and concentration changes. Based on this, the dynamics in both liquid and crystalline phases will be discussed later. During analysis, the slope change in the second region (i.e., during the crystallization process as measured by the terahertz beam) at different frequencies was extracted as well as the corresponding time in the region. In general, the larger the gradient was, the faster the crystals grew, and the less time it spent in this stage of the process. In most experiments, the gradient of the linear fit was positive for a frequency of 1.6 THz and negative at lower frequencies, reflecting that, during the crystallization, absorption at the frequency of the vibrational peak of the crystalline feature increased while that of liquid phase dropped.

Figure 5a clearly shows the different behavior of the absorption for the peak at 1.6 THz compared to other frequencies. While the absorption at 1.6 THz (triangles) increased during crystallization, it decreased for lower frequencies (dots and diamonds). The spread was higher at higher concentrations, meaning that a faster crystallization was more likely to result from more supersaturated solutions of MgSO_4_.

If less time was spent in the second region, i.e., the crystal growth rate was increased, the higher the absolute gradient in that region at all frequencies would be. This was shown in Figure 5b. For better clarity, the slope during the phase at which the crystals grew into the field of view of the THz-TDS system was shown at only 1 THz, where the signal-to-noise ratio was the largest. The slope in the second region of the data at 1 THz was plotted against the time the crystals took to fully cover the field of view of the spectrometer. The shown slope was negative, because the absorption decreased at 1 THz when crystals appeared.

Figure 5c shows the relationship between the duration of the middle region with the temperature at which the experiments were performed. While crystal growth through the field of view of the spectrometer seemed to take around 4 min at temperatures between 3.5 to 5 °C, the spread was larger at temperatures above 6 °C. In one extreme case, it took almost 40 min for the crystals to fully cover the field of view. In most other cases, it took between 2 to 12 min, independent of concentration. Finally, Figure 5d shows how much the absorption at 1.0 THz changed with time at different temperatures and concentrations.

Combining Figure 5a and Figure 5c, it was concluded that, based on the results from our experiments presented here, both a higher initial concentration and elevated temperature above 6 °C made the crystal growth more erratic indicated by a wider spread of the data.

### Calibrated Local Concentration and Hydration Shell

Terahertz spectra are inherently temperature-dependent. As discussed above, both a decrease in MgSO_4_ concentration and an increase in temperature yield a higher absorption coefficient. Therefore, if the data are corrected for temperature variations, all changes that are observed in the absorption coefficient are directly linked to structural changes of the probed sample volume.

To eliminate temperature effects, a calibration procedure previously established^23^ was followed. By measuring the absorption of liquid mixtures of varying concentrations at different temperatures, a calibration curve had previously been determined. This allowed the calculation of the concentration of a solution of unknown concentration at arbitrary temperatures. In purely liquid samples, the calibration procedure resulted in the actual concentration for solutions. However, the emergence of crystalline features affected the spectra, and in this case, the liquid phase absorption was calculated at frequencies furthest away from the peak of the crystalline feature at 1.6 THz.

**Figure 6:**
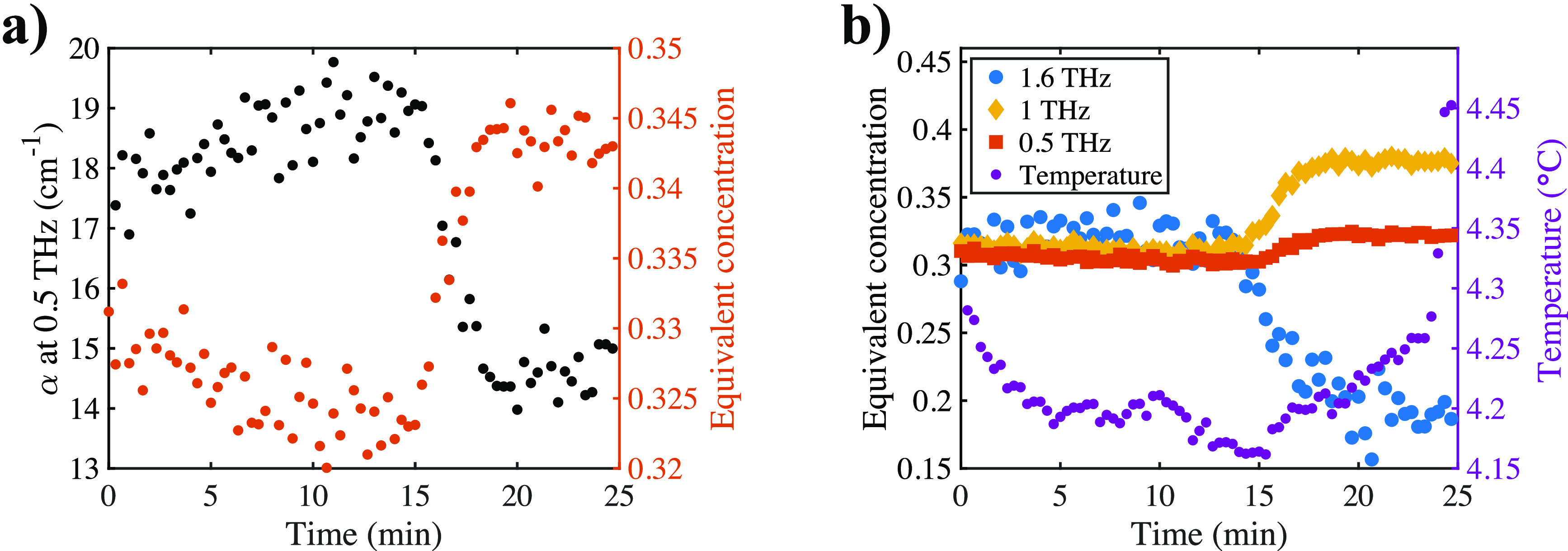
Illustration of using the calibration method to calculate the liquid phase solute concentration based on measured absorption at various frequencies. (a) Absorption α at 0.5 THz (black) and corresponding calculated liquid phase solute concentration over time (red). (b) Liquid phase solute concentration calculated at 0.5 THz (red squares), 1.0 THz (orange diamonds), and 1.6 THz (blue dots). Right: The temperature throughout the measurement. It was stable and stayed within 0.1 °C of the set point until the temperature control was turned off after crystallization.

An example of converting temperature-dependent data into the temperature-independent equivalent concentration is shown in Figure 6. Instead of the slowly rising absorption before crystals reached the field of view that was observed in Figure 4, the concentration decreased. This complemented the information gained only by analyzing the absorption coefficient and yielded an explanation of the changes in the spectra during crystallization as follows.

At the beginning of the experiment, both the terahertz spectra and visual analysis confirmed the absence of MgSO_4_ crystals located in the center of the cell. Once nucleation occurred, typically not in the center of the cuvette but near one end of the cell, a local increase in water concentration was observed in the terahertz spectra due to the increase in water concentration immediately adjacent to the growing crystals as magnesium and sulfate ions crystallized into the MgSO_4_ heptahydrate form. This caused a slight increase of the absorption coefficient, corresponding to a lower MgSO_4_ concentration measured in the center of the cell.

MgSO_4_ in solution is surrounded by a hydration shell whose absorption is markedly different from that of bulk water.^44^ This was demonstrated by calculating a theoretical absorption coefficient based on the known absorption coefficient of pure water (α_water_) and that of anhydrous MgSO_4_ and that of MgSO_4_·7H_2_O (α_crystal_), neglecting the effect of a larger hydration shell. A series of MgSO_4_ aqueous solutions with a range of concentrations were measured, and a difference between the measured (α_solution_) and the calculated (α_ideal mixture_) absorption was consistently observed.
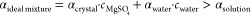
1

**Figure 7:**
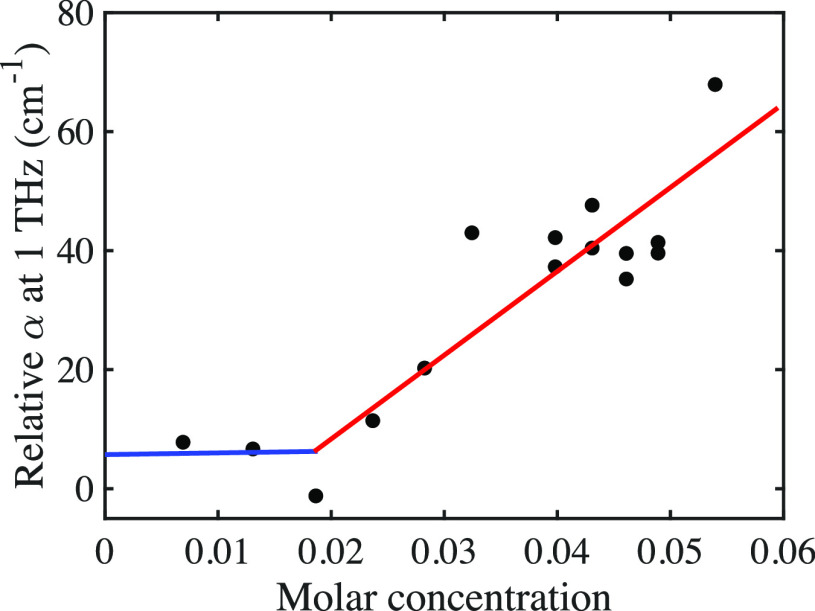
Relative absorption calculated and plotted against molar concentration of MgSO_4_. The experimental error is on the order of 4 cm^–1^, because the absolute absorption coefficients between different measurements and concentrations are compared. Within one measurement, the absolute error is lower (e.g., 1–2 cm^–1^). The lines are drawn to guide the eye and are not intended to be indicative of a physical model.

This difference was calculated with eq 1 for a number of measurements and is shown in Figure 7 for anhydrous MgSO_4_, where *c*_MgSO_4__ represents the molar concentration of anhydrous MgSO_4_ or MgSO_4_·7H_2_O of the solutions in the corresponding case. Calculated values for both anhydrous MgSO_4_ and MgSO_4_ heptahydrate are shown in Table S2 in the SI. Changes were subtle below 0.02 molar concentration and increased steadily above in both the cases of anhydrous MgSO_4_ and MgSO_4_ heptahydrate.

The calculated theoretical absorption excluding the effect of the hydration shell was larger than that of the measured absorption, indicating that the hydration shell surrounding MgSO_4_ had a lower absorption coefficient than the bulk water that it replaced, which is in line with expectation, as the dipoles in the hydration shell tend to exhibit slower relaxation behavior.^45−47^ The results also inferred that the hydration shell encompassed more than the seven water molecules that form part of the MgSO_4_ heptahydrate crystal because of the observed difference between the measured and calculated absorption. This is in line with other observations that also found extended hydration shells when probing samples with THz-TDS.^44^

An increase of the overall absorption coefficient at 0.5 and 1.0 THz as seen in experiments when crystals grew hence corresponded to water being expelled from the hydration shells into the bulk phase when the crystals formed. The bulk aqueous phase is pushed toward the field of view sampled by THz-TDS during the growth of the crystals before the crystals themselves enter the field of view of the terahertz beam. Therefore, the growth of MgSO_4_ heptahydrate, which started at one end of the cell, increased the local concentration of bulk water in the center of the cell, where it was probed with THz-TDS. This explained the initial slight increase in absorption that was observed at both 0.5 and 1.0 THz, given that the absorption coefficient of bulk water is much higher than that of the solution mixed with MgSO_4_ or the heptahydrate.

Once the crystals reached the center of the cell, the absorption at 0.5 and 1 THz decreased (see Figure 4), as the probed sample became more ordered, and thereby the VDOS was depleted, while the absorption at 1.6 THz increased as the peak emerged. On the other hand, the liquid phase concentration seemed to increase at 0.5 and 1.0 THz when crystals started to grow in the field of view of THz-TDS. This was in line with a decrease of the hydration shell size and a potentially denser liquid in the area of forming crystals that was probed with THz-TDS.^3^ The absorption coefficient at the peak at 1.6 THz clearly increased, and this effect was accompanied by a decrease in MgSO_4_ concentration. Once the crystals covered the center of the cell and the system reached an equilibrium state, the changes at all frequencies became subtle again.

However, the calculated liquid phase concentration was not quantitatively valid at frequencies close to crystal features, as no rigorous method has yet been developed within the framework presented^23^ to systematically account for peak effects to the baseline, and the calibration curve was determined from the experimental data of a series of samples in the liquid phase only. Others have used a multifeature model^48^ that could be used in the future to explore this further, but in the present work, we were not relying on the modeling of peaks and wanted to avoid making further assumptions.

To examine further the influence of the crystalline feature to the data collected at other frequencies, the previous procedure for calculating concentration was applied inversely, i.e., the known and frequency-independent calculated concentrations were used to calculate the equivalent absorption if it was fully liquid (α_liquid_). Of the three frequencies described here, the data at 0.5 THz were the least affected by the crystalline feature, since that frequency was the furthest away from the feature at 1.6 THz. Therefore, the concentration calculated from it was being used as the basis to calculate α_liquid_.

**Figure 8:**
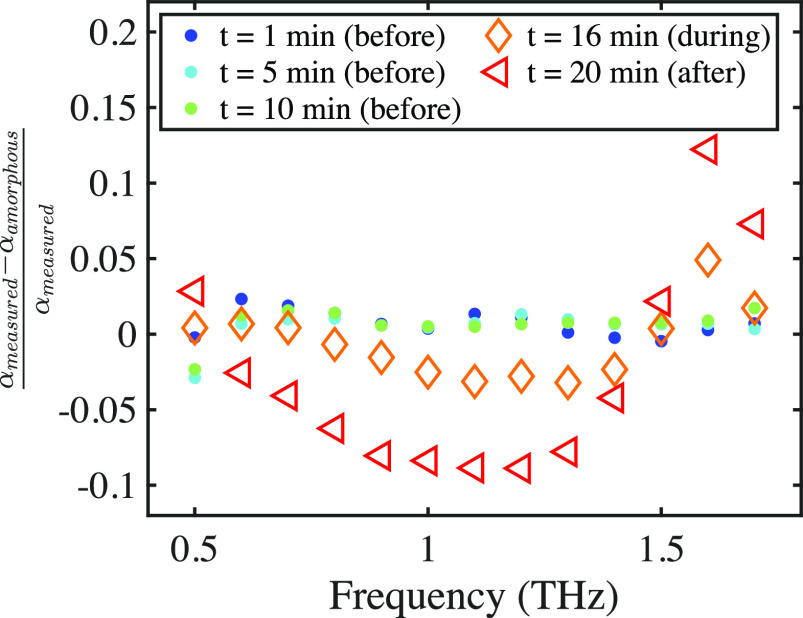
Relative changes in α when comparing the measured to the calculated purely amorphous absorption before, during, and after crystallization.

The relative difference compared to the measured absorption is plotted in Figure 8 for different experimental stages. Before crystals were observed in the field of view, the relative difference was close to zero for all frequencies. During crystal growth into the field of view, however, the relative difference increased between 1.5 to 1.7 THz and decreased 0.6 to 1.4 THz. This effect became even stronger once crystallization was complete. Maxima of the relative difference were found at 1 and 1.6 THz, while the difference decreased toward lower frequencies. This showed that while the peak only seemed to impact a relatively narrow frequency range between 1.5 and 1.7 THz, the effects of crystallization are still strongly observed at 1.0 THz. The spectral change was directly visible: depletion of the VDOS below 1.5 THz and appearance of a peak above. It should be noted that while current results focus on crystal growth into field of view of the spectrometer, nucleation itself has not yet been observed directly. This will be the focus of future work, possibly by observing very subtle spectral changes.

### Dissolution Observed

All the crystallization experiments were performed and monitored at a constant temperature, and once both visual and spectral analysis confirmed that the crystallization was completed, the system was slowly heated up. Meanwhile, it was also observed that crystals started to dissolve at elevated temperatures. Therefore, further measurements were carried out to study this phenomenon systematically.

With the well-controlled heating component, an experimental heating rate of 0.2 °C min^–1^ was determined to ensure a constant temperature change in the crystallization cell. Faster heating might have led to a temperature difference between the circulating water and the inside of the cell, while slower heating rates (although possible) prolonged the experiment. With the chosen heating rate, crystal dissolution was observed within a reasonable experimental time frame. However, an accurate dissolution temperature was not measured, because hysteresis effects related to the kinetics of crystallization and dissolution have to be taken into account.

**Figure 9:**
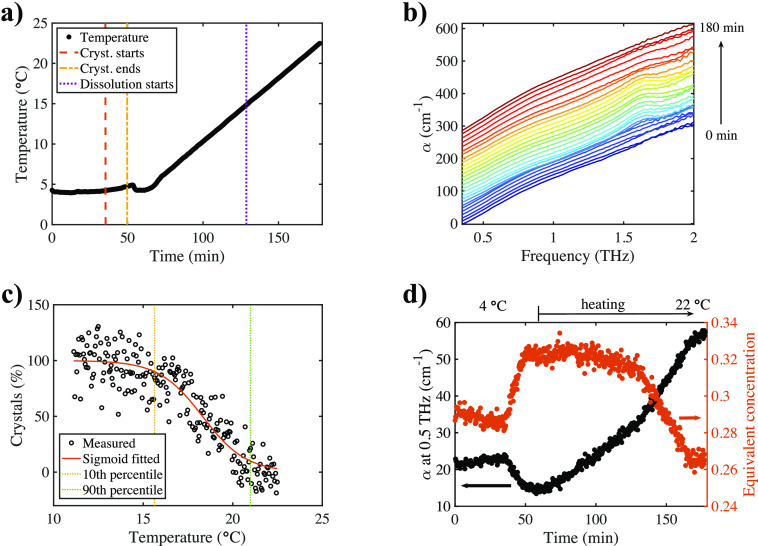
Crystal dissolution analysis. (a) Temperature profile during the experiment. Vertical lines denote changes in the spectra. (b) Terahertz spectra acquired throughout the experiment (liquid–semicrystalline–liquid). Subsequent spectra are offset by 10 cm^–1^. (c) Dissolution of crystals observed with visual analysis. Dissolving started at a temperature shortly below 16 °C and was completed just above 20 °C. At a heating rate of 0.2 °C min^–1^, this process took about 34 min to complete. (d) α at 0.5 THz and corresponding calculated concentration over time. After initial crystallization, the temperature was steadily increased up to room temperature.

The temperature profile over time is shown in Figure 9a, and the times when characteristic changes occurred in the spectra are highlighted with vertical lines. These agreed well with the times extracted from the images acquired by the camera. When the temperature was increased steadily once crystals grew completely, crystal dissolution was observed both visually as the percentage of crystals decreased drastically in the cell (Figure 9c) as well as with THz-TDS resulting in the disappearance of the crystalline feature at higher temperatures (Figure 9b). This was also investigated by utilizing the calculation of liquid phase concentrations to remove the temperature effect from the spectra (Figure 9d).

The calculated concentration stayed mostly constant once crystals had formed until around 130 min after the beginning of the experiment, which coincided with the first observation of crystal dissolution in the camera images. Because heat was constantly being added to the system by the heat of dissolution of magnesium sulfate and the temperature increased steadily, the equilibrium concentration of MgSO_4_ in the vicinity of the crystal features varied, because the saturation point changed with temperature. Therefore, the crystals dissolved slowly while the surrounding liquid was approaching the point of local saturation upon increasing the temperature. Opposite to crystal growth, dissolution resulted in an enlargement of the hydration shells accompanied by an increase of the absorption at 0.5 and 1.0 THz and a decrease at 1.6 THz. The calculated concentration however decreased at 0.5 THz.

## Conclusion

THz-TDS was used to study the crystallization process of MgSO_4_·7H_2_O. The emergence and disappearance of the spectral feature at 1.6 THz indicated the growth or dissolving of crystals in the field of view of the spectrometer (validated by image analysis), while the change of the baseline reflected the behavior of solvent. This is useful for investigating solvation dynamics and the behavior of molecular species at phase boundaries.

The absorption at three frequencies was investigated in particular, and the process clearly showed three stages. Experiments at three concentrations and in the temperature range of 4 to 9 °C suggested that both a higher initial concentration and elevated temperature above 6 °C were likely to result in a more erratic crystal growth. The faster the crystals grew through the field of view of the spectrometer, the higher was the change in absorption at all frequencies. The temperature effect on terahertz spectra was addressed as outlined previously,^21^ leading to the calculation of an equivalent liquid phase concentration. In addition, changes in the absorption coefficient were correlated with the composition and size of the hydration shell surrounding the salt ions.

The results covered here are from experiments where the crystals grew into an area probed by terahertz radiation. Therefore, the onset of nucleation was not observed directly. The focus of ongoing work is to trigger nucleation at desired locations (e.g., in the center of the cell) so that the investigation can be extended from that of crystal growth to that of nucleation. The current setup is designed for operating temperatures between 4 to 9 °C, and this range can be extended further with simple adjustments. Therefore, this technique can be applied to investigate a wide range of crystalline and semicrystalline systems, thereby offering an interesting perspective of low-frequency motions of multiphase systems.

## Supporting Information Available

Figures of PXRD patterns of anhydrous MgSO_4_, MgSO_4_·H_2_O (with simulated pattern), MgSO_4_·6H_2_O, and MgSO_4_·7H_2_O (with simulated pattern), figure of comparison of refractive index and absorption coefficient, figure of the metal block and the crystallization cell, schematic of the experimental setup, table of concentration overview for different solid-state samples, table of data for Figure 7, table of solid-state DFT optimized atomic positions for the asymmetric unit cell of MgSO_4_·7H_2_O; unit cell orientations for magnesium sulfate heptahydrate mode animations (from ss-DFT) (PDF)
Magnesium sulfate heptahydrate mode animations (ZIP)
Computationally optimised structure of magnesium sulphate heptahydrate (CIF)

## Supplementary Material

cg2c00352_si_002.zip

cg2c00352_si_003.cif
