# Supplementary material for: In Situ Observation of the Structure of Crystallizing Magnesium Sulfate Heptahydrate Solutions with Terahertz Transmission Spectroscopy
Source: Cryst Growth Des. 2022 May 20;22(6):3961–72. doi: 10.1021/acs.cgd.2c00352 (PMC9165030; doi:10.1021/acs.cgd.2c00352)
Supplement: Supplementary file 1 — cg2c00352_si_001.pdf [file cg2c00352_si_001.pdf]

Supplementary Information for In-Situ Observation of the Structure of Crystallising Magnesium Sulfate Heptahydrate Solutions with Terahertz Transmission Spectroscopy

Qi Li[a#], Johanna Kölbel [a#], Margaret P. Davis[b], Timothy. M. Korter[b], Andrew D. Bond[c], Terence L. Threlfall[d], and J. Axel Zeitler[a*]

[a]Department of Chemical Engineering and Biotechnology, University of Cambridge, Philippa Fawcett Drive, Cambridge, CB3 0AS, UK.

[b]Department of Chemistry, Syracuse University, Syracuse, NY, USA.

[c]Yusuf Hamied Department of Chemistry, University of Cambridge, Lensfield Road, Cambridge, CB2 1EW, UK.

[d]Department of Chemistry, University of Southampton, Southampton, SO17 1BJ, UK.

[#]Contributed equally to this work

[*]Corresponding author

Email: jaz22@cam.ac.uk

Table of contents

Figure S1: PXRD pattern of anhydrous $MgSO_4$ of two different suppliers and comparison with simulated pattern. ..……………………………………………….3

Table S1: Concentration overview for different solid-state samples. ………………...4

Figure S2: PXRD pattern of $MgSO_4{\cdot}H_2O$ and comparison with simulated pattern. Additional peaks are marked with * and indicate the presence of an additional minor phase. …………………………………………………………………………….5

Figure S3: PXRD pattern of $MgSO_4{\cdot}6H_2O$ for comparison. ………………………...6

Figure S4: PXRD pattern of $MgSO_4{\cdot}7H_2O$ (bottom) and comparison with simulated

pattern (top). …………………………………………………………………………..7

Table S2: Data for Figure 7. …………………………………………………………..8

Table S3: Solid-state DFT optimized atomic positions in fractional coordinates for the asym- metric unit cell of $MgSO_4 \cdot 7H_2O$, space group P212121 with a=11.9503 Å, b=11.7913 Å, and c=6.6789 Å. …………………………………………………………9

Figure S5: Comparison of refractive index in arbitrary units and absorption coefficient (inset) for the same measurement as shown in Figure 4. Absolute values are arbitrary because the reference was acquired in air, introducing a phase shift that has not been corrected. Right: Change in refractive index during the same measurement. For clarity, only every second spectrum is shown.………………………………………10

List S1: Unit cell orientations for magnesium sulfate heptahydrate mode animations (from ss-DFT)……………………………………..………………………11

Figure S6: Left: Rendering of the metal block and the encompassed crystallisation cell. Right: Schematic of the crystallisation cell, lengths are in mm...………………12

Figure S7: Schematic of the setup……………………………………………………13

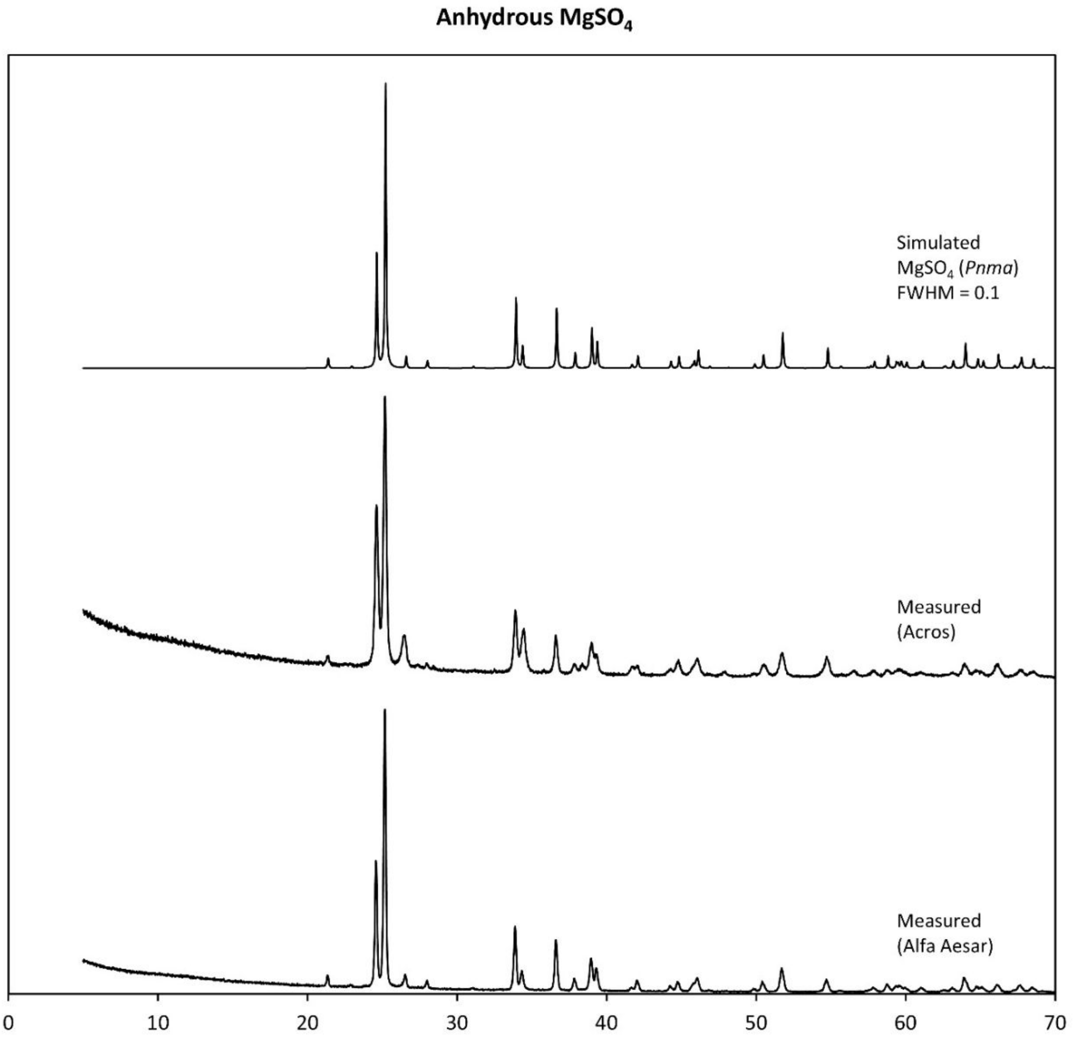

Figure S1: PXRD pattern of anhydrous $MgSO_4$ of two different suppliers and comparison with simulated pattern.

Table S1: Concentration overview for different solid-state samples.

Sample	Concentration
Anhydrous $MgSO_4$ 97% (AcrosOrganics)	7.5% w/w
Anhydrous $MgSO_4$ 99.5% (AlfaAesar)	7.5% w/w
$MgSO_4$ monohydrate 97% (Sigma-Aldrich)	6.3% w/w
$MgSO_4$ heptahydrate 98% (Sigma-Aldrich)	2.5% w/w
$MgSO_4$ heptahydrate crystallised at room temperature from solution	2.5% w/w

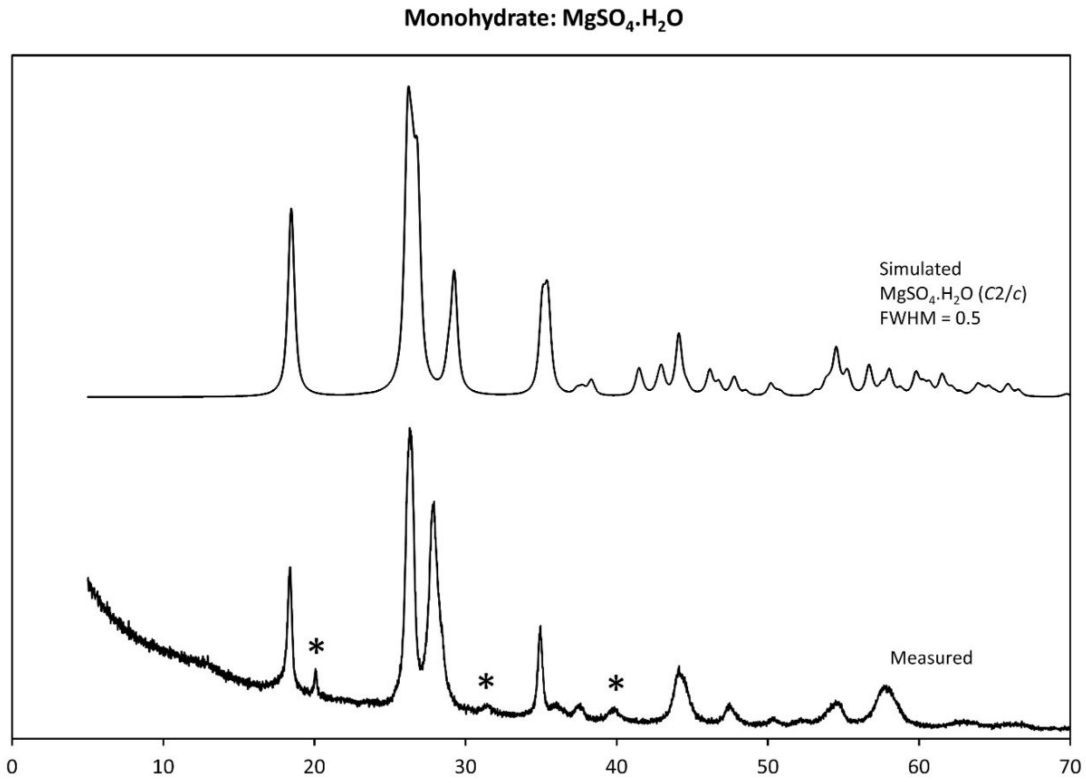

Figure S2: PXRD pattern of $MgSO_4{\cdot}H_2O$ and comparison with simulated pattern. Additional peaks are marked with * and indicate the presence of an additional minor phase.

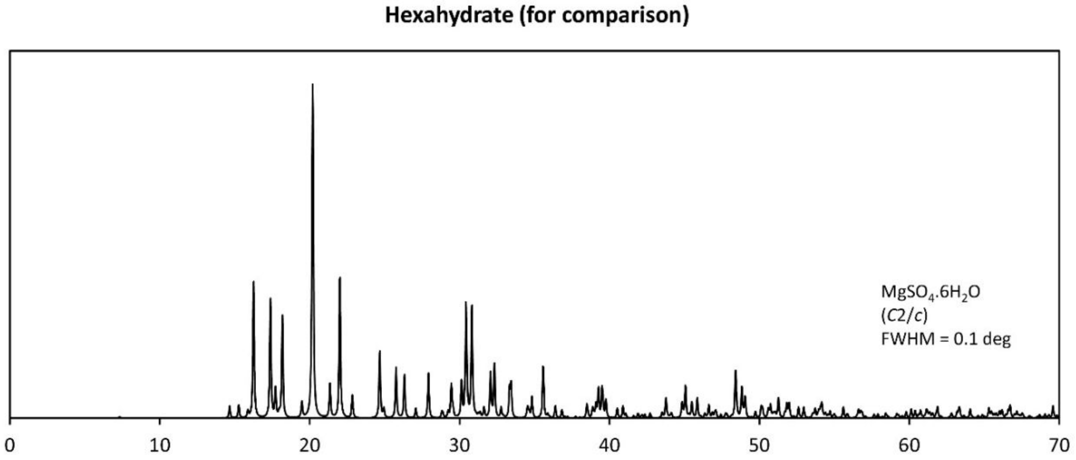

Figure S3: PXRD pattern of $MgSO_4 \cdot 6H_2O$ for comparison.

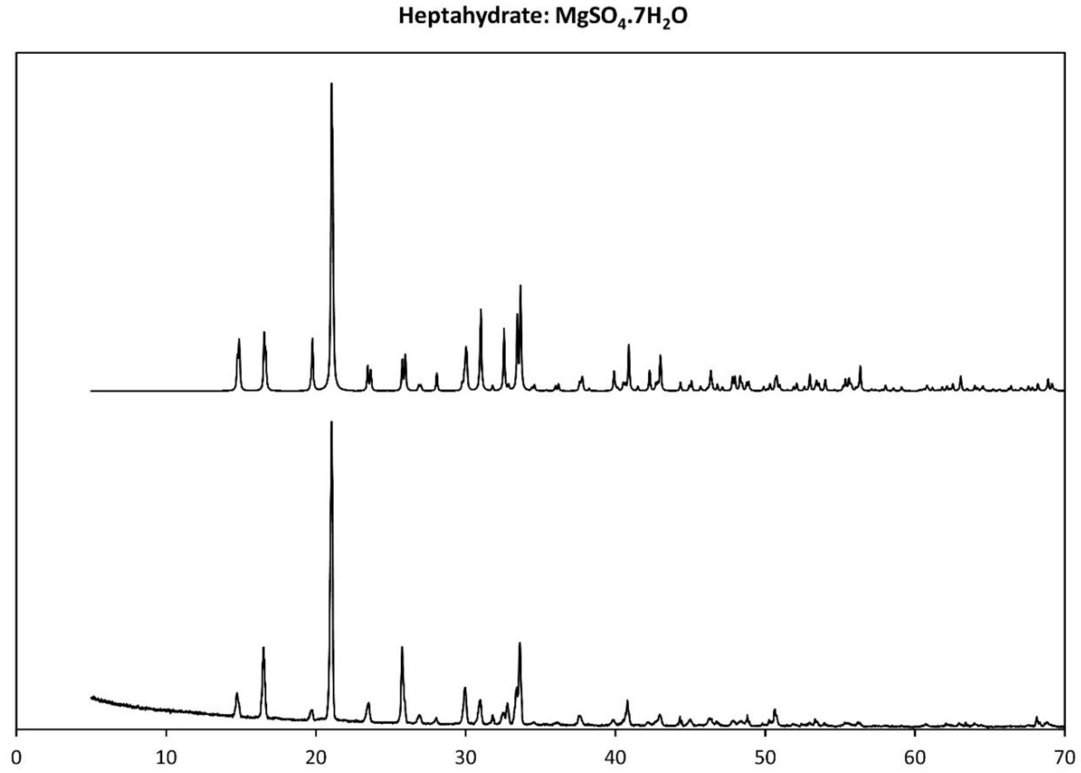

Figure S4: PXRD pattern of $MgSO_4 \cdot 7H_2O$ (bottom) and comparison with simulated pattern (top).

Table S2: Data for Figure 7.

Molar conc. ($MgSO_4$)	Relative abs. (cm^{-1})	Molar conc. ($MgSO_4 \cdot 7H_2O$)	Relative abs. (cm^{-1})
0.07	7.80	0.007	7.96
0.013	6.68	0.014	6.85
0.019	-1.21	0.021	-1.15
0.024	11.42	0.028	11.27
0.028	20.26	0.035	19.81
0.032	43.00	0.042	42.18
0.04	37.29	0.055	35.55
0.04	42.19	0.055	40.45
0.043	40.44	0.062	38.17
0.043	47.64	0.062	45.37
0.046	39.54	0.068	36.7
0.046	35.24	0.068	32.4
0.049	39.59	0.074	36.15
0.049	41.39	0.074	37.95
0.054	67.92	0.087	63.21

Table S3: Solid-state DFT optimized atomic positions in fractional coordinates for the asym- metric unit cell of $MgSO_4 \cdot 7H_2O$, space group P212121 with a=11.9503 Å, b=11.7913 Å, and c=6.6789 Å.

Atom (Atomic Number)	X/A	Y/B	Z/C
12	4.14869E-01	1.04554E-01	3.79462E-02
16	-2.79618E-01	1.72530E-01	4.99110E-01
8	-3.23214E-01	5.71696E-02	4.25480E-01
8	-1.49699E-01	1.76100E-01	4.84627E-01
8	-3.17371E-01	1.92850E-01	-2.83242E-01
8	-3.28556E-01	2.70179E-01	3.65637E-01
8	2.59751E-01	1.74195E-01	-4.11522E-03
8	4.69374E-01	2.52213E-01	1.94024E-01
8	4.61883E-01	1.70888E-01	-2.34092E-01
8	-4.26816E-01	4.20697E-02	7.43259E-02
8	3.69239E-01	-4.51565E-02	-1.11311E-01
8	3.55893E-01	3.98353E-02	3.01333E-01
8	-4.96859E-01	4.32095E-01	-6.17005E-02
1	2.31020E-01	2.26061E-01	9.93225E-02
1	2.31941E-01	1.98406E-01	-1.35500E-01
1	4.18230E-01	2.76919E-01	3.01968E-01
1	-4.55480E-01	2.51110E-01	2.56265E-01
1	4.20454E-01	2.25309E-01	-3.15915E-01
1	-4.57130E-01	1.80605E-01	-2.56082E-01
1	-3.83393E-01	5.12019E-02	1.98792E-01
1	-4.00576E-01	-2.81914E-02	1.28248E-02
1	3.62300E-01	-1.13260E-01	-2.72502E-02
1	4.15624E-01	-6.19240E-02	-2.31127E-01
1	2.76061E-01	3.27032E-02	3.32270E-01
1	4.00486E-01	1.30305E-04	4.03426E-01
1	4.36772E-01	4.80464E-01	-4.74693E-02
1	4.86532E-01	3.62055E-01	1.27172E-02

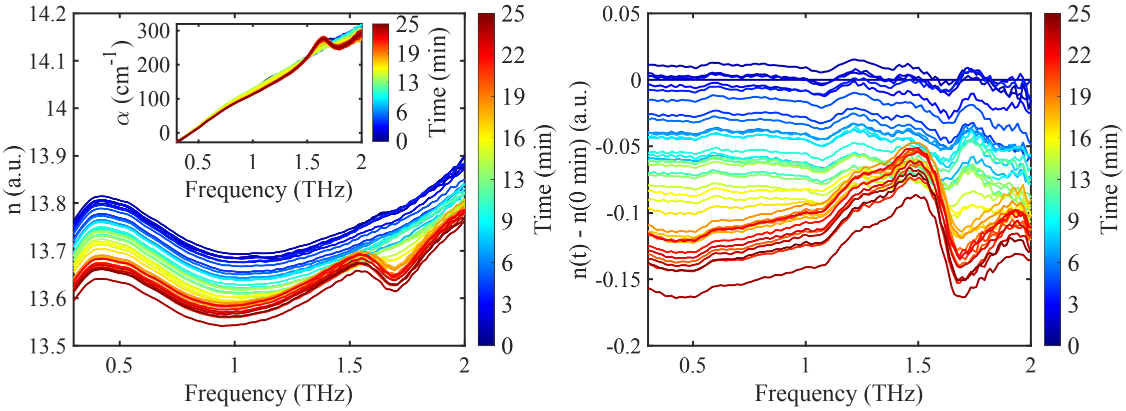

Figure S5: Left: Comparison of refractive index in arbitrary units and absorption coefficient (inset) for the same measurement as shown in Figure 4. Absolute values are arbitrary because the reference was acquired in air, introducing a phase shift that has not been corrected. Right: Change in refractive index during the same measurement. For clarity, only every second spectrum is shown.

List S1: Unit cell orientations for magnesium sulfate heptahydrate mode animations (from ss-DFT)

All animations were generated using the Jmol software (Jmol: an open-source Java viewer for chemical structures in 3D. http://www.jmol.org/).

a. 1.74 THz, 57.88 cm^{-1}, down c axis (horizontal a, vertical b)
b. 1.93 THz, 64.23 cm^{-1}, down a axis (horizontal c, vertical b)
c. 1.96 THz, 65.49 cm^{-1}, down c axis (horizontal a, vertical b)
d. 2.40 THz, 79.89 cm^{-1}, down c axis (horizontal a, vertical b)
e. 2.78 THz, 92.64 cm^{-1}, down a axis (horizontal c, vertical b)
f. 2.83 THz, 94.38 cm^{-1}, down c axis (horizontal a, vertical b)

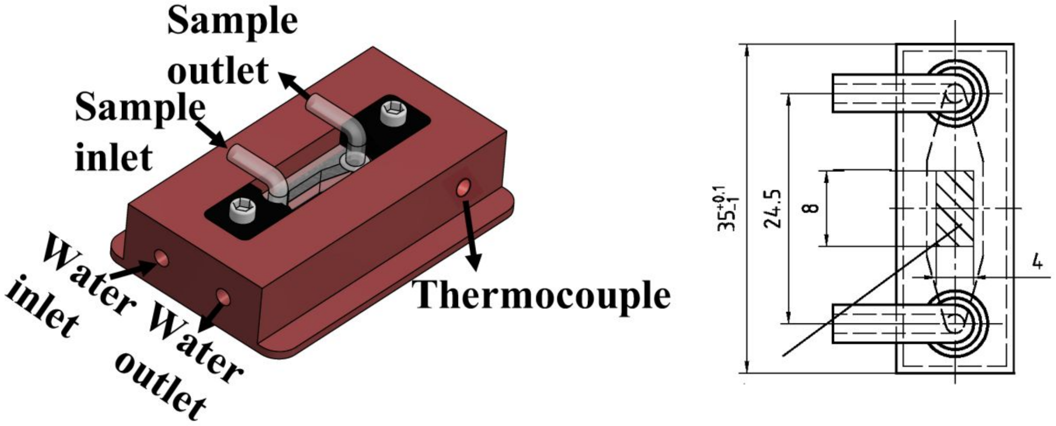

Figure S6: Left: Rendering of the metal block and the encompassed crystallisation cell. Right: Schematic of the crystallisation cell, lengths are in mm. Modified from ref 24. Copyright 2022 IEEE.

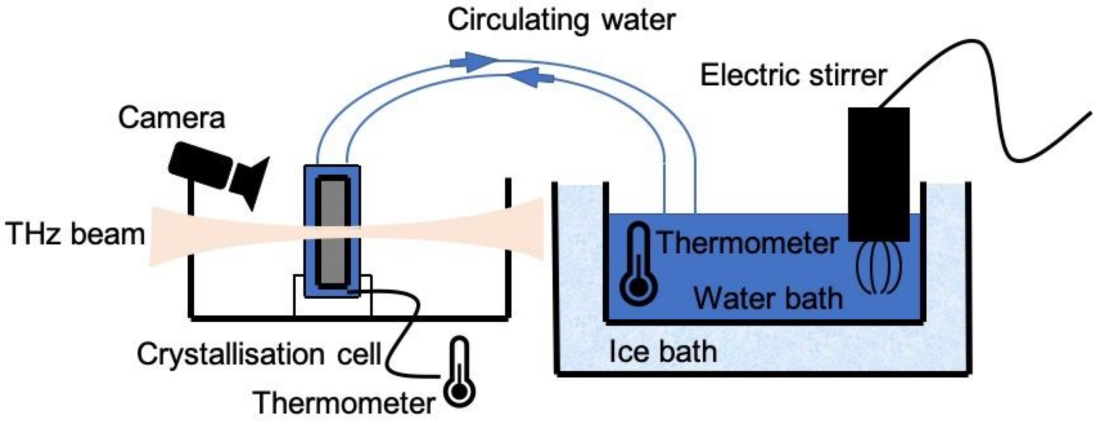

Figure S7: Schematic of the setup. Modified from ref 24. Copyright 2022 IEEE
